# Epidemiological and clinical features of pediatric COVID-19

**DOI:** 10.1186/s12916-020-01719-2

**Published:** 2020-08-06

**Authors:** Cheng-Xian Guo, Li He, Ji-Ye Yin, Xiang-Guang Meng, Wei Tan, Guo-Ping Yang, Tao Bo, Jun-Ping Liu, Xin-Jian Lin, Xiang Chen

**Affiliations:** 1 Center of Clinical Pharmacology, the Third Xiangya Hospital, Central South University, Changsha, 410013 Hunan China; 2 Department of Pediatrics, The Third Xiangya Hospital, Central South University, Changsha, 410013 Hunan China; 3 Department of Clinical Pharmacology, Xiangya Hospital, Central South University, Changsha, 410008 Hunan China; 4 Laboratory of Cardiovascular Disease and Drug Research, Zhengzhou No. 7 People’s Hospital, Zhengzhou, 450016 Henan China; 5 Department of Neonatology, Maternal& Child Health Hospital of Guangxi Zhuang Autonomous Region, Nanning, 53003 Guangxi Zhuang Autonomous Region China; 6 Institute of Ageing Research, Hangzhou Normal University School of Medicine, Hangzhou, 311121 Zhejiang China; 7 Department of Immunology, Monash University School of Medicine, Melbourne, Victoria 3004 Australia; 8 Key Laboratory of Gastrointestinal Cancer (Fujian Medical University), Ministry of Education, Fuzhou, 350122 Fujian China; 9 Department of Dermatology, Xiangya Hospital, Central South University, Changsha, 410008 Hunan China

**Keywords:** Pediatric, Coronavirus, SARS-CoV-2, COVID-19, Epidemiology, Clinical features

## Abstract

**Background:**

COVID-19 is an extremely severe infectious disease. However, few studies have focused on the epidemiological and clinical characteristics of pediatric COVID-19. This study conducted a retrospective review of the epidemiological and clinical features of COVID-19 in children.

**Methods:**

A retrospective study was conducted on children with a definite diagnosis of COVID-19 in mainland China using the web crawler technique to collect anonymous COVID-19 updates published by local health authorities.

**Results:**

Three hundred forty-one children aged 4 days to 14 years with a median age of 7 years were included. Sixty-six percent of pediatric patients were infected via family members with COVID-19. The median incubation period was 9 days (interquartile range, 6 to 13). Asymptomatic cases accounted for 5.9%, of which 30% had abnormal chest radiologic findings. A majority of pediatric COVID-19 cases showed mild to moderate clinical features, and only a few developed severe or critical diseases (0.6% and 0.3%, respectively). Fever (77.9%) and cough (32.4%) were the predominant presenting symptoms of pediatric COVID-19. The pediatric patients had fewer underlying diseases and complications than adults. The treatment modalities for pediatric COVID-19 patients were not as complex as those of adult COVID-19 patients. The overall prognosis of pediatric COVID-19 was benign with a decent recovery. The median time from onset to cure was 16 days (interquartile range, 13 to 21).

**Conclusions:**

Compared to adults, COVID-19 in children has distinct features of epidemiology and clinical manifestations. The findings from this study might help to guide the development of measures to prevent and treat this ongoing global pandemic.

**Trial registration:**

Chinese Clinical Trial Registry (chictr.org.cn) identifier: ChiCTR2000030464.

## Background

At the end of 2019, a sudden outbreak of novel coronavirus pneumonia in Wuhan, China, brought a series of calamities to both Chinese society and global communities [1]. The International Committee on Taxonomy of Viruses (ICTV) names the new coronavirus as severe acute respiratory syndrome coronavirus 2 (SARS-Cov-2), and World Health Organization (WHO) designates the pandemic disease caused by SARS-Cov-2 as coronavirus disease 2019 (COVID-19) [2, 3]. Although COVID-19, severe acute respiratory syndrome (SARS), and Middle East respiratory syndrome (MERS) are all caused by coronavirus and can be manifested with severe respiratory distress, COVID-19 has its own epidemiological and clinical features [4]. In adults, COVID-19 has the characteristics of a long incubation period, strong infectivity, atypical clinical symptoms, and high mortality in the elderly [5–7]. In view that people at any age are susceptible to COVID-19 and it has spread widely around the world, COVID-19 may affect human health for a long period of time [1, 6, 7]. Therefore, understanding of the epidemiological and clinical features of COVID-19 will help to control the spread and improve the curative rate of this pandemic disease.

Compared to adults, there are relatively few studies on pediatric COVID-19 [8–10]. In particular, the epidemiological and clinical characteristics of COVID-19 in children aged 0–14 years are yet to be fully defined. With the current rapid worldwide spread of SARS-CoV-2 infection, the number of pediatric patients with COVID-19 is expected to increase significantly or has already been rising remarkably [9]. Therefore, defining the epidemiological and clinical features of the disease in large cohorts of pediatric patients is an urgent need. In this report, we conducted a retrospective review of COVID-19 features in 341 pediatric patients with ages between 0 and 14 years with the overall goal of providing data that could help in the development of guidelines for the prevention and treatment of pediatric COVID-19.

## Methods

### Data collection

This retrospective review was conducted in children aged 0–14 years with a definite diagnosis of COVID-19 from local health authorities between January 15, 2020, and March 15, 2020, in mainland China. Based upon the method previously described by Wei et al. and Xu et al. with slight modifications, we collected anonymous data on COVID-19 as epidemic updates were publicized by local health authorities using the web crawler technique [8, 11]. Web crawler is a program or script that automatically crawls web information according to certain rules [11]. In this study, we used the web crawler technology implemented by combining the Python language with Mysql database to collect COVID-19 data via the following procedures: (1) determination of the source of data collection through the website and portal of each local health administration department in China; (2) data interface analysis and data capture on information acquired from the website of each case release by imitating the hypertext transfer protocol_GET requests (HTTP_GET); (3) data cleaning by removing noise and irrelevant data, merging the data from multiple data sources into a data store, and then converting the original data into a form suitable for data mining; and (4) data warehousing by storing the cleaned data in the Mysql database. The diagnostic criteria of COVID-19 were based on “Diagnosis and Treatment Protocol for 2019-nCoV” [9, 12]. The patients were eligible for inclusion if they met the following criteria: (1) younger than 14 years old and (2) at least two consecutive positivity of SARS-CoV-2 by the nucleic acid test. Those patients older than 14 years old and whose information on age and gender were not available were excluded from the study. Two independent reviewers screened the subjects for eligibility. The data on gender, age, geographic location, and epidemiological history was collected together with clinical features on presenting symptoms, diagnosis, and therapeutic outcome. The study protocol was approved by the Institutional Review Board (AAHRPP-accredited) of the Third Xiangya Hospital of Central South University and registered with the Chinese Clinical Trial Registry (www.chictr.org, registration number: ChiCTR2000030464).

### Data processing

SPSS19 and R 3.6.1 were used for data analysis. The incubation period was defined as the time from exposure to the onset of symptoms and expressed as a median and inter-quartile range. A descriptive statistical analysis was performed, and ratios or percentages were compared.

## Results

### Epidemiological characteristics of pediatric COVID-19

Clinical profiles collected from a total of 7097 patients with a definite diagnosis of COVID-19 from local health authorities were used. Patients older than 14 years of age or with an undefined age and gender were excluded. Three hundred forty-one children aged from 4 days to 14 years with a median age of 7 years were included in the study (Table 1 and Additional file 1: Table S1). A female-to-male gender ratio of 1.2:1 (183 boys and 158 girls) was found (Table 1).

**Table 1:** Epidemiological characteristics of pediatric COVID-19

Classification	Characteristic
Included patient number	341
Age, median (range)	7 years (4 days to 14 years)
Gender ratio	
Male/female (%)	183/158 (1.2)
Exposure to the source of transmission No.%	
Family members with COVID-19	210/318 (66%)
Non-family members with COVID-19	10/318 (3.1%)
History of exposure to epidemic area	92/318 (28.9%)
Indefinite history of exposure	6/318 (1.9%)
Data unavailable	23/341 (6.7%)
Incubation period (days)	85
Median (range)	9 (0–20)
Quartile (Q1–Q3)	6–13

As shown in Table 1, family clustering was a major transmission route for pediatric COVID-19. There were 210 patients (66%) who were infected by close contact with family members with COVID-19. Among the 210 pediatric patients, two neonates born to mothers with COVID-19 were tested positive for SARS-CoV-2 on day 4 and day 5 after birth, respectively. Ten cases (3.1%) were infected via close contact with non-family members diagnosed with COVID-19. Ninety-two patients (28.9%) had a history of exposure in an epidemic area, including residence in and around Wuhan or a recent trip to Wuhan or its vicinity. Six (1.9%) children had an undefined history of exposure since they had not taken any trips to epidemic zones or had no contact with definitely diagnosed cases or with individuals having a recent history of visiting Wuhan or adjoining areas. Data were invalid for 23 (6.7%) children.

Eighty-five (25.2%) children had a defined incubation time, which is the time frame from exposure to an infectious source to the onset of symptoms (Table 1). The median incubation period was 9 days (ranging 0 to 20 days), and the interquartile range was 6 to 13 days.

### Clinical characteristics of pediatric COVID-19

Twenty of 341 (5.9%) children were asymptomatic (with no overt clinical symptoms and negativity of SARS-CoV-2 virus infection by the nucleic acid test). Computed tomography (CT) indicated that 6 (30%) patients among 20 asymptomatic children had abnormal chest radiograph (Table 2). The disease severity was categorized according to “Diagnosis and Treatment Protocol for 2019-nCoV” [9, 12]. Three hundred and eighteen patients were diagnosed as mild (clinical symptoms without any imaging evidence of pneumonia) or moderate (fever and respiratory tract symptoms with imaging evidence of pneumonia) severity accounting for 93.3% of all cases, 2 (0.6%) had severe (an onset of respiratory distress with acute hypoxia) symptoms, and 1 (0.3%) was classified as critical case (an onset of respiratory failure and shock) (Table 2).

**Table 2:** Clinical characteristics of pediatric COVID-19

Classification	Characteristic (No., %)
**Clinical diagnosis**	341
Asymptomatic	20 (5.9%)
With abnormal chest radiograph	6 (30%)
Mild/moderate	318 (99.3%)
Severe	2 (0.6%)
Critical	1 (0.3%)
**Clinical symptoms**	136
Fever (37.3~40 °C)	106 (77.9%)
Cough	44 (32.4%)
Lacrimation	6 (4.4%)
Stuffy nose	3 (2.2%)
Sneezing	1 (0.7%)
Sore throat	3 (2.2%)
Dyspnea	3 (2.2%)
Nausea & vomiting	4 (2.9%)
Diarrhea	6 (4.4%)
Conjunctivitis	1 (0.7%)
Dizziness and headache and myalgia	3 (2.2%)
Fatigue	3 (2.2%)
Body discomfort	3 (2.2%)
**Coexisting disorders**	136
Congenital heart	2 (1.5%)
**Complications**	136
Heart failure	1 (0.7%)
Myocardial injury	6 (4.4%)
Liver injury	3 (2.2%)
Acute respiratory distress syndrome/shock/acute kidney injury	1 (0.7%)
**Treatment**	62
Antiviral treatment	33 (53.2%)
TCM	27 (43.5%)
Antibiotic treatment	17 (27.4%)
Nutritional support therapy	17 (27.4%)
Symptomatic treatment	17 (27.4%)
Interferon therapy	7 (11.3%)
Immunoglobulin therapy	4 (6.5%)
Psychological intervention	2 (3.2%)
Not taking medicine	1 (1.6%)
Glucocorticoid therapy	1 (1.6%)
Mechanical ventilation	1 (1.6%)
**Clinical outcome**	
Death	0/341
**Time from onset to cure** (days)	218
Median (range)	16 (6–39)
Quartile (Q1–Q3)	13–21

One hundred and thirty-six had a detailed description of clinical symptoms (Table 2). The symptoms that the pediatric COVID-19 patients presented included fever (77.9%) with body temperature ranging from 37.3 to 40 °C, cough (32.4%), lacrimation (4.4%), stuffy nose (2.2%), sneezing (0.7%), sore throat (2.2%), dyspnea (2.2%), nausea and vomiting (2.9%), diarrhea (4.4%), conjunctivitis (0.7%), dizziness/headache/myalgia (2.2%), fatigue (2.2%), and physical discomfort (2.2%) (Table 2).

Children with COVID-19 had fewer underlying diseases (Table 2). Among the pediatric patients in our study, two (1.5%) had concomitant congenital heart disease. Severe complications were also rare in the pediatric patients recruited in our study, of whom 1 (0.7%) developed heart failure and 6 (4.4%) and 3 (2.2%) had the signs of myocardial injury and liver injury, respectively (Table 2). In the critical case that has been reported as a case study, the complications included shock, acute kidney injury, and acute respiratory distress syndrome [13].

Both symptomatic and asymptomatic children with SARS-CoV-2 infection were hospitalized while asymptomatic patients may not require any therapy. For symptomatic patients with abnormal chest radiograph, therapeutic management generally included supportive therapy and antiviral treatment. As shown in Table 2, the study retrieved treatment information from 62 patients, in which 33 (53.2%) patients received antiviral therapy (lopinavir and ritonavir tablets), 27 (43.5%) received traditional Chinese medicine (Lianhua Qingwen capsules), 17 (27.4%) received antibacterial therapy (azithromycin), 17 (27.4%) received nutritional support therapy, 17 (27.4%) received symptomatic treatment (e.g., fever reduction, cough relief, myocardial nutrition, liver protection, diarrhea relief), 7 (11.3%) received interferon-α therapy, 4 (6.5%) received oxygen therapy, 4 (6.5%) received immunoglobulin therapy, and 2 (3.2%) received a psychological intervention. The critically ill patient in this study received comprehensive treatment including glucocorticoids and mechanical ventilation [13].

The overall prognosis of children with COVID-19 was benign with a decent recovery. There was no mortality in this pool of pediatric COVID-19 (Table 2). In 218 cases including three children with severe or critical clinical type, the median time from onset to cure was 16 days (ranging from 6 to 39), and the interquartile range was 13 to 21 days.

### Comparison of characteristics of pediatric COVID-19 with adult COVID-19

As shown in Table 3, we compared the differences between pediatric COVID-19 and adult COVID-19. Pediatric COVID-19 cases were mostly transmitted in family clustering while adults can be infected in multiple routes. It is worth noting that there may have been maternal-fetal vertical transmission of SARS-CoV-2 in newborns. The median and interquartile range of the incubation period in pediatric COVID-19 were 9 days and 6–13 days, respectively; the median and interquartile range in adult COVID-19 were 4 days and 2–7 days, respectively. 5.9% of the pediatric patients were asymptomatic whereas approximately 1% of the adult COVID-19 patients were asymptomatic. Pediatric patients with COVID-19 were mainly mild/moderate, while adults, especially the elderly, tended to be more severe/critical. The clinical symptoms of COVID-19 in adults were more complicated than those in children although fever and cough were the main clinical manifestations for both adults and children. Compared with adults, children with COVID-19 had fewer comorbid conditions and complications. The treatment modalities for adult COVID-19 patients were more complicated than those for children with COVID-19. Children with COVID-19 recovered well whereas COVID-19 prognosis in adults was relatively worse with respect to the clinical outcome.

**Table 3:** Comparison of the characteristics of COVID-19 between children and adults

Classification	Children	Adults [5, 6, 14]
**Exposure to source of transmission**	Family cluster (may have maternal-fetal vertical transmission)	Multiple ways (local residents of Wuhan, recently been to Wuhan, contacted with people from Wuhan and wildlife)
**Incubation period (days)**		
Median (range)	9 (0–20)	4 (0–24)
Quartile (Q1–Q3)	6–13	2–7
**Clinical diagnosis**		
Asymptomatic	5.9%	1%
Abnormal chest radiograph	30%	No data available
Mild/moderate	99.3%	81%
Severe	0.6%	14%
Critical	0.3%	5%
**Clinical symptoms and signs**		
Fever	77.9%	43.1%
Cough	32.4%	67.7%
Other	Lacrimation, stuffy nose, sneezing, sore throat, dyspnea, nausea and vomiting, diarrhea, conjunctivitis, dizziness and headache and myalgia, fatigue, body discomfort	Conjunctival congestion, nasal congestion, headache, sore throat, dyspnea, sputum production, fatigue, hemoptysis, shortness of breath, nausea or vomiting, diarrhea, myalgia or arthralgia, chill, throat congestion, tonsil swelling, enlargement of lymph nodes, rash
**Coexisting disorders**	Congenital heart	Hypertension, diabetes, coronary heart disease, hepatitis B infection, chronic obstructive pulmonary disease, chronic renal diseases, immunodeficiency, cancer, cerebrovascular diseases, neurological manifestations
**Complications**	Heart failure, myocardial injury, liver injury, acute respiratory distress syndrome, shock, acute kidney injury	Septic shock, acute respiratory distress syndrome, acute kidney injury, acute cardiac injury, disseminated intravascular coagulation, rhabdomyolysis, pneumonia, secondary infection
**Treatment**	Symptomatic treatment, antiviral treatment, TCM, antibiotic treatment, nutritional support therapy, interferon therapy, immunoglobulin therapy, glucocorticoid therapy, mechanical ventilation	Symptomatic treatment, antiviral treatment, antibiotic treatment, antifungal medications, corticosteroids, immunoglobulin therapy, TCM, interferon therapy, noninvasive ventilation, invasive mechanical ventilation, extracorporeal membrane oxygenation (ECMO), continuous renal replacement therapy (CRRT)
**Clinical outcomes**		
Death	0	2.3%
**Time from onset to cure (days)**		
Median (range)	16 (6–39)	No data available
Quartile (Q1–Q3)	13–21	No data available

## Discussion

The current rapid global spread of SARS-CoV-2 infection prioritizes our intense efforts to identify effective preventive strategies and develop optimal medical management. Although there is relatively ample information available for adult COVID-19 patients, our knowledge and analysis of the epidemiology and clinical characteristics of pediatric COVID-19 is quite limited. In this context, we performed a retrospective review of COVID-19 in children under 14 years old to assess the epidemiological and clinical features of the pediatric COVID-19. This systematic review of pediatric patients with COVID-19 showed that children with COVID-19 were mainly infected via family clustering and had a long incubation period. The majority of patients infected by SARS-CoV-2 presented as asymptomatic or mild/moderate disease. The most frequent clinical manifestations were fever and cough. Children with COVID-19 had rare comorbid conditions and few severe complications. The medical management for the pediatric COVID-19 patients mainly included supportive therapy and antiviral treatment. In general, the pediatric patients with COVID-19 had a good prognosis.

Pediatric patients acquired COVID-19 by a clear route of transmission that included close contact with family members with COVID-19 or a history of exposure to epidemic areas, or both. In our study, 66% of the pediatric patients were diagnosed after their family members were confirmed to be infected with SARS-CoV-2. In particular, two neonates were infected with SARS-CoV-2, followed by their mothers being confirmed with COVID-19. Although previous studies including 19 newborns have downplayed the possibility of maternal-fetal vertical transmission of SARS-CoV-2 [15, 16], we cannot rule out such a potential risk. A study reported by Zeng et al. also found that 3 of 33 newborns born to pregnant women infected with SARS-CoV-2 were diagnosed with COVID-19 [17]. Irrespective of insufficient evidence of vertical transmission, there was definitely a high neonatal risk of SARS-CoV-2 infection if a mother contracted COVID-19. Furthermore, our study indicates that the source of infection could not be traced for some cases of pediatric COVID-19. The epidemiological profiles of 1.9% of children included remain unknown since they had never visited any epidemic zone, contacted anyone from an epidemic zone, or been around anyone with a definitive COVID-19 diagnosis. That may add a new layer of complexity for the transmission of COVID-19 in children and may highlight the importance of strategies such as minimizing close contact with strangers even for children.

Our study also demonstrates that the median and interquartile range of the incubation period for pediatric COVID-19 were 9 days and 6–13 days, compared to 4 days and 2–7 days for adults with COVID-19. This difference might be explained by the fact that children’s immune system is far from mature and may respond to pathogens differently to adults. Furthermore, younger children, especially at pre-school age, may not clearly describe their own health conditions and contact history, which could contribute to the delay in seeking medical attention and making the diagnosis. Regardless of underlying causes, the result that the incubation period of COVID-19 was longer in children than it was in adults might implicate that parents should monitor children more closely when the family members have COVID-19, and a long medical observation period for children exposed to SARS-CoV-2 should be warranted.

The prevalence of pediatric asymptomatic infection was estimated at 5.9% in this study, which was higher than 1% in the study by Wu et al. on adult patients [14]. Unexpectedly, some cases of asymptomatic children had abnormal radiologic findings. The percentage of asymptomatic children with abnormal chest radiographic presentation was as high as 30%. Although an abnormal chest radiograph did not predict the symptoms and severity of pediatric COVID-19 patients, the presence of pulmonary lesions in asymptomatic patients may suggest the need for medication to reduce pulmonary inflammation. At present, there is no report of pulmonary imaging lesions in asymptomatic adult patients. The presenting clinical symptoms of pediatric COVID-19 were often atypical. Fever and cough were the main symptoms that could be accompanied by gastrointestinal symptoms such as nausea, vomiting and diarrhea, and other symptoms like sneezing, stuffy nose, sore throat, dizziness, headache, myalgia, and conjunctivitis. COVID-19 symptoms in children generally followed a similar pattern in adults, albeit much less severe and more atypical [6]. In this study, we found that pediatric patients had fewer underlying diseases and complications than adult patients. One child with COVID-19 was comorbid with congenital heart disease, and severe complication such as heart failure, myocardial injury, or liver injury was observed in one, six, or three children, respectively. The underlying diseases of adult COVID-19 patients included hypertension, diabetes, and coronary heart disease while many patients, especially severe patients, may have the complications of septic shock, acute respiratory distress syndrome, and acute kidney injury, etc.

Although there are no clear guidelines for the treatment of pediatric COVID-19, our study suggests that the treatment measures for pediatric COVID-19 patients were not as complex as that of adult COVID-19 patients, but even relatively simple. The treatment modalities for children with COVID-19 were mainly composed of antiviral therapy, traditional Chinese medicine, empirical antibiotic treatment, nutritional support therapy, and symptom reliefs. The time from the onset to recovery in children with COVID-19 was 6 to 39 days, with a median of 16 days and an interquartile of 13 to 21 days. The prognosis of children with COVID-19 was decent. However, we still cannot relax the stringency of monitoring of affected children, and we should be alert to the possibility of aggravation caused by delayed treatment. The critical patient included in this study is likely to result from delayed treatment [13]. It may be worth noting that there is a significant patient overlap between this study and the one recently reported by Dong et al. [9] due to the fact that both studies used the same pool of pediatric COVID-19 cases. However, what our study adds is the detailed clinical findings including clinical symptoms, therapeutic management, and prognosis of pediatric COVID-19 in addition to analysis of the epidemiological characteristics of COVID-19 in children that have also been defined by the prior study [9].

This study has several limitations. The research only covered a brief 2-month period with observational design and retrospective nature. The data was obtained from local China health authorities thus unable to compare the epidemiological and clinical data from US and European studies in children with COVID-19. We were also unable to correlate viral burden with clinical severity due to the limitation of SARS-CoV-2 virus nucleic acid test per se. Lastly, our study encountered a problem of missing some clinical information, particularly detailed treatment strategies. However, it should be recognized that due to the low incidence of COVID-19 in children, our analysis is in the forefront to clarify the epidemiological and clinical lack of knowledge on pediatric COVID-19. Moreover, to our knowledge, the sample size of this study represents a relatively large and comprehensive survey of the characteristics of children with COVID-19.

## Conclusions

In summary, compared to adults, COVID-19 in children has distinct features of epidemiology and clinical manifestations. The findings from this study might help to formulate strategies and guidelines for prevention and treatment of pediatric COVID-19.

## Supplementary information

**Additional file 1: Supplementary Table S1.** The distribution and clinical type of confirmed cases in mainland China.

## Data Availability

All data is available by application to the study authors.

## Abbreviations

COVID-19: Coronavirus disease 2019
ICTV: The International Committee on of Taxonomy of Viruses
MERS: Middle East respiratory syndrome
SARS: Severe acute respiratory syndrome
SARS-CoV-2: Severe acute respiratory syndrome coronavirus 2
WHO: World Health Organization
