# Supplementary material for: Epidemiological and clinical features of pediatric COVID-19
Source: BMC Med. 2020 Aug 6;18:250. doi: 10.1186/s12916-020-01719-2 (PMC7408975; doi:10.1186/s12916-020-01719-2)
Supplement: Supplementary file 1 — Additional file 1: Supplementary Table S1. The distribution and clinical type of confirmed cases in mainland China. [file 12916_2020_1719_MOESM1_ESM.docx]

**Table S1. The distribution and clinical type of confirmed cases in mainland China**

| Geographic location | Total (n) | Clinical diagnosis | | |
| --- | --- | --- | --- | --- |
|  |  | Asymptomatic | Mild/ moderate | Severe /Critical |
| Anhui | 28 | 1 | 27 | - |
| Beijing | 6 | - | 6 | - |
| Chongqing | 4 | - | 4 | - |
| Fujian | 1 | - | 1 | - |
| Gansu | 2 | - | 2 | - |
| Guangdong | 64 | 1 | 63 | - |
| Guangxi | 14 | 1 | 13 | - |
| Guizhou | 10 | - | 10 | - |
| Hainan | 3 | - | 3 | - |
| Hebei | 12 | 1 | 11 | - |
| Heilongjiang | 4 | - | 4 | - |
| Henan | 29 | - | 28 | 1 |
| Hubei | 31 | 3 | 26 | 2 |
| Hunan | 19 | 1 | 18 | - |
| Jiangsu | 12 | - | 12 | - |
| Jiangxi | 8 | - | 8 | - |
| Jining | 3 | - | 3 | - |
| Liaoning | 1 | 1 | - | - |
| Ningxia | 1 | 1 | - | - |
| Qinghai | 2 | - | 2 | - |
| Tianjin | 2 | - | 2 | - |
| Shaanxi | 8 | - | 8 | - |
| Shanxi | 5 | 1 | 4 | - |
| Shandong | 33 | 5 | 28 | - |
| Shanghai | 4 | - | 4 | - |
| Sichuan | 18 | 2 | 16 | - |
| Yunnan | 7 | - | 7 | - |
| Zhejiang | 10 | 2 | 8 | - |
|  | 341 | 20 | 318 | 3 |
